# The increased ability to present citrullinated peptides is not unique to HLA-SE molecules: arginine-to-citrulline conversion also enhances peptide affinity for HLA-DQ molecules

**DOI:** 10.1186/s13075-016-1153-4

**Published:** 2016-11-03

**Authors:** Arieke S. B. Kampstra, Jurgen van Heemst, Antonis K. Moustakas, George K. Papadopoulos, Tom W. J. Huizinga, René E. M. Toes

**Affiliations:** 1 Department of Rheumatology, Leiden University Medical Center, Leiden, The Netherlands; 2 Department of Organic Farming and Food Technology, Technological Educational Institute of Ionian Islands, Argostoli, Greece; 3 Laboratory of Biochemistry and Biophysics, Faculty of Agricultural Technology, Epirus Institute of Technology, Arta, Greece; 4 Department of Rheumatology C1-R, Leiden University Medical Center, Albinusdreef 2, PO Box 9600, Leiden, 2300 RC The Netherlands

**Keywords:** Rheumatoid arthritis, ACPA, Shared epitope, Smoking, Citrulline

## Abstract

**Background:**

Presentation of citrullinated neo-epitopes by HLA-DRB1 molecules that carry the shared epitope (SE) sequence was proposed to explain the association between HLA and seropositive RA. Although it is shown that several HLA-DRB1-SE molecules display enhanced binding affinities for citrullinated ligands, the ability of other HLA molecules to present citrullinated epitopes has not been investigated in a systematic manner. To better understand the HLA-RA connection, we aimed to investigate if the enhanced capacity to present arginine-to-citrulline-converted peptides is unique for HLA-SE alleles.

**Methods:**

We selected four HLA molecules (one HLA-DR and three HLA-DQ molecules) that could potentially prefer citrulline over arginine residues in specific pockets and in addition two HLA-SE alleles as a method validation control. The affinity of peptides containing arginine/citrulline residues at positions interacting with the various peptide-binding pockets was compared by HLA class II peptide affinity assays.

**Results:**

Pocket 4 of HLA-DRB1*04:04 and -DRB1*04:05 displayed a preference for citrulline over arginine, a property found in other pockets as well. HLA-DRB1*03:01 did not display an enhanced affinity for peptides containing a citrulline. In contrast, several peptide-binding pockets of the analyzed HLA-DQ molecules showed enhanced affinities for citrulline compared to arginine residues: i.e., pockets 4, 6, 7, and 9 of HLA-DQ2 and pockets 1, 6, and 9 of HLA-DQ7 and HLA-DQ8.

**Conclusions:**

Arginine-to-citrulline conversion of peptides can also enhance the binding affinity for non-HLA-SE molecules. Hence the capacity to present citrullinated neo-epitopes is not confined to HLA-SE molecules, opening the possibility that also other HLA molecules could potentiate a possible breach of T cell tolerance toward citrullinated antigens.

## Background

Rheumatoid arthritis (RA) patients can be divided in two distinct subsets based on the presence of anti-citrullinated protein antibodies (ACPA) [1, 2]. These autoantibodies target proteins that have undergone posttranslational modifications converting positively charged arginine to uncharged citrulline residues [3].

The most important risk factor for ACPA-positive RA is the human leukocyte antigen (HLA) class II locus [2]. This locus contains many genes, including genes encoding the beta chain of HLA-DR (*HLA-DRB1*) and the alpha and beta chain of HLA-DQ (*HLA-DQA1* and *HLA-DQB1*) that are inherited together in haplotypes. In 1987, it was proposed that the risk of developing ACPA-positive RA is explained by the HLA-DRB1 locus, in particular by those alleles that carry the sequence QKRAA, QRRAA or RRRAA in positions 70–74 of the DRB1 chain, also known as the shared epitope (SE) motif [4, 5]. About 80 % of ACPA+ RA patients carry “HLA-SE” alleles, illustrating that these alleles are highly prevalent, but not required to develop seropositive disease [2].

The ligand-binding groove of HLA class II molecules is composed of both the alpha and beta chains and is involved in the presentation of peptides to CD4^+^ T cells. The ligands accommodated by HLA class II molecules vary in length, but the part that interacts most strongly with this groove is 9 amino acids (AA) in length. The amino acids within this part, also referred to as the peptide core, are anchored within the groove in peptide-binding pockets. These pockets accommodate the side chains of peptide residues 1, 4, 6, 7, and 9 and are respectively named: pocket 1, 4, 6, 7, and 9 [6].

The amino acid residues that shape the peptide-binding pockets are highly polymorphic, thereby dictating the preferences for particular amino acids that can be accommodated in the pockets of different HLA class II alleles. In this way, the combined characteristics of the peptide-binding pockets shape the repertoire of ligands presented by the HLA molecule. The amino acids forming the SE motif are part of pocket 4 of the HLA-DRB1 molecule [4, 6]. It was proposed that the SE motif associates with risk because the positively charged SE residues would prevent the accommodation of positively charged arginine residues while favoring the accommodation of uncharged citrulline residues in pocket 4 [7]. The conversion of arginine to citrulline could thereby result in the presentation of a peptide that would otherwise not be present [7]. Indeed, several studies have shown that pocket 4 of HLA-SE alleles HLA-DRB1*01:01, *04:01, *04:04, and *10:01 prefers citrulline over arginine residues [7–9].

It was postulated that this preference for citrulline over arginine residues would be unique to HLA-SE alleles, but this was never analyzed [7]. We aimed to study if non-HLA-SE alleles could also present citrullinated peptides with enhanced affinity.

## Methods

### Cell lines

Lysates of Epstein-Barr virus (EBV)-transformed lymphoblastoid B cell lines were used as a source for HLA-DQ/DR molecules: BOLETH (DQA1*03:01/DQB1*03:02), BSM (DQA1*03:01/DQB1*03:02), DUCAF (DRB1*03:01/DQA1*05:01/DQB1*02:01), JSM (DQA1*03:02/DQB1*03:01), and YOT (DRB1*04:05). Cells were maintained in Iscove’s modified Dulbecco’s medium (IMDM) (Lonza Bioresearch, Basel, Switzerland) supplemented with 8 % heat-inactivated fetal calf serum (FCS) (Gibco; Life Technologies, Carlsbad, CA, USA), 1 % penicillin, streptomycin, and GlutaMAX (Invitrogen, Carlsbad, CA, USA).

### Peptides

Peptides were synthesized according to standard Fmoc (N-(9-fluorenyl)methoxycarbonyl) chemistry using a SyroII peptide synthesizer (MultiSynTech, Witten, Germany). The integrity of the peptides was verified using reverse-phase high-performance liquid chromatography (HPLC) and mass spectrometry (MS). For binding studies on HLA-DR4 molecules we used macrophage migration inhibition factor (MIF)_33-47_ KPPQ**Y**IA**V**H**VV**P**D**QL and apolipoprotein B-100 (ApoB)_2588-2603_ PDF**I**VP**L**T**DL**R**I**PSVQ, for HLA-DR3 myoglobin peptide_138-148_ L**F**RK**D**I**AA**K**Y**K, for HLA-DQ2 major histocompatibility complex (MHC) class I alpha_50-62_ PW**I**EQ**E**G**PE**F**W**DQ, and for HLA-DQ7 and HLA-DQ8 herpes simplex virus (HSV) 2 peptide VP16_432-444_ VD**M**TP**A**D**AL**D**D**FD. The anchors are depicted in bold. These peptides were previously described in the context of the studied HLA molecules [10–12]. To assess arginine or citrulline accommodation, peptides with an arginine or a citrulline at the indicated anchor positions were generated. As negative control peptides, we used the following peptides that do not bind to the respective HLA molecules: for HLA-DR4 and -DR3 we used AAAAKAAAAA, for HLA-DQ2 AKPFPQPEAPYKA, and for HLA-DQ7.3 and HLA-DQ8 AADTNRWSKMDAA (data not shown).

### HLA class II competitive peptide-binding assay

Peptide-binding assays were performed, as previously described [13]. In short, cell lysates from HLA class II homozygous B-lymphoblastoid cell lines were incubated on SPV-L3- (anti-HLA-DQ)- or B8.11.2- (anti-HLA-DR)-coated (10 μg/ml) FluoroNunc 96-well plates at 4 °C overnight. Test peptides in the range of 0 to 300 μM were mixed with a fixed concentration (0.6 μM) of biotinylated indicator peptide and added to the wells. Bound indicator peptide was detected using Europium-streptavidin (PerkinElmer, Boston, MA, USA) and measured in a time-resolved fluorometer (PerkinElmer, Wallac Victor2). IC50 values were calculated based upon the observed binding of the test peptide against the fixed concentration indicator peptide. The IC50 value depicts the concentration of test peptide required for a loss of 50 % of the indicator peptide signal. IC50 values greater than 300 μM were classified as non-detectable binding.

### Statistical analysis

Wilcoxon’s signed-rank test was used to assess differences in IC50 values between citrulline- and arginine-containing peptides. *P* values below 0.05 were considered to be statistically significant.

### Model structures

Model structures of peptides citrullinated in specific pockets were obtained by molecular simulation as previously described [13]. Essentially, the crystal structure ls9v.pdb was used for modeling of HLA-DQ2 peptide complexes. The Discover Suite (programs Insight II and Discover) of Accelrys Software Inc. (San Diego, CA, USA, release of 2005) was used on a Silicon Graphics Fuel (Mountain View, CA, USA) instrument, using a standard minimization approach. Occasionally, runs were performed on a Silicon Graphics Octane instrument with previous releases of the same software obtaining very similar results. Minimizations were carried out at pH 5.4 (endosomal pH), the same pH used in the peptide-binding assays. Figures are drawn using the WebLabViewer v3.5 and DSViewerPro software of Accelrys, the latter currently freely available on the web. The coordinates of the minimized structures are available to interested researchers upon request to Dr. G. K. Papadopoulos (gpapadop@teiep.gr).

## Results

### Presentation of arginine and citrulline residues by HLA-DR3

**Fig. 1:**
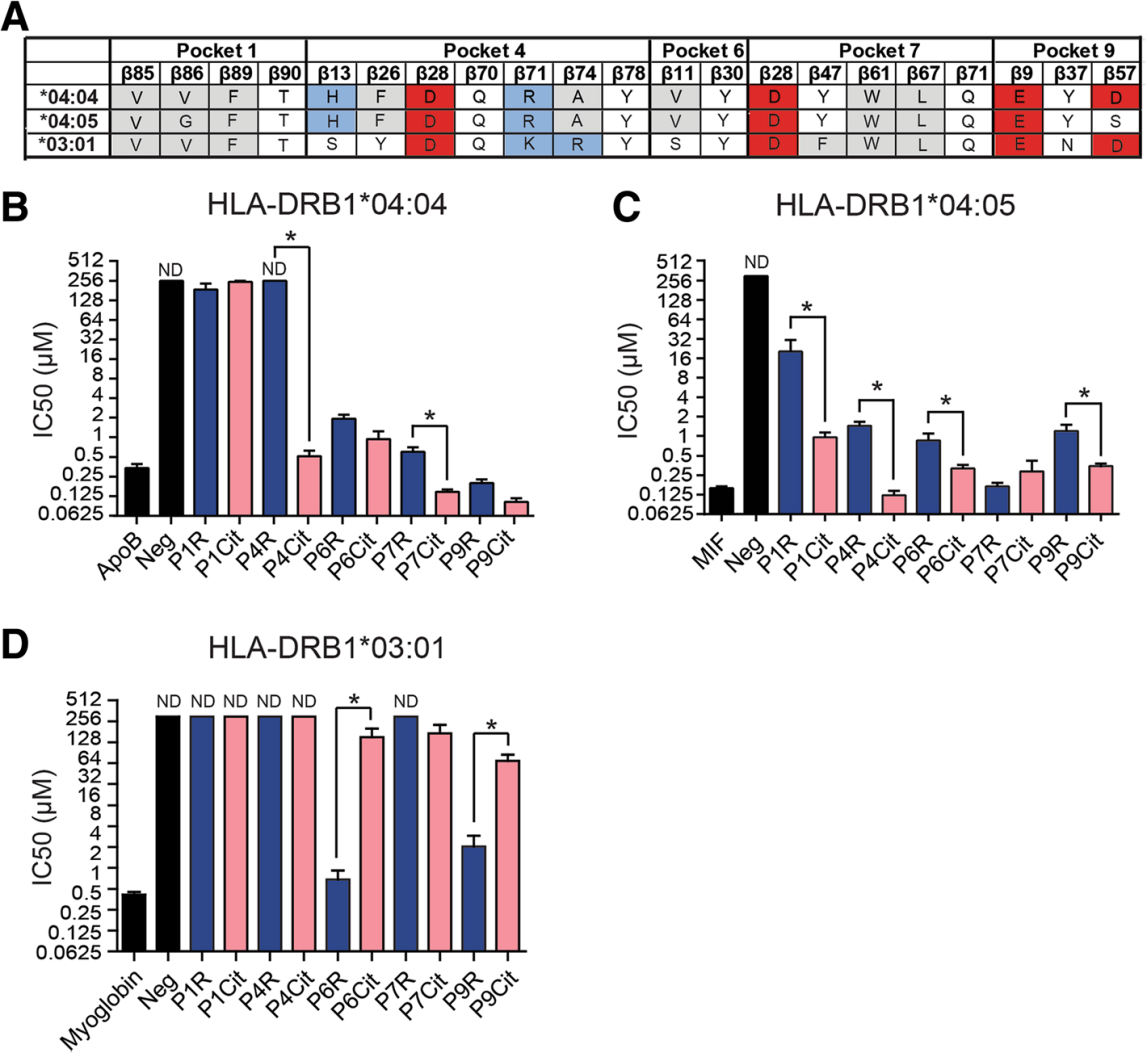
Accommodation of citrulline and arginine residues by HLA-DR4 and HLA-DR3 molecules. **a** Schematic representation of the peptide-binding pockets of HLA-DR4 and HLA-DR3. Amino acid (AA) residues are color coded according to their properties (*white* = hydrophilic, *gray* = hydrophobic, *red* = acidic, *blue* = basic). **b** Competitive binding of a biotin-labeled ApoB peptide with an unlabeled ApoB peptide or ApoB variants with arginine or citrulline residues in p1, p4, p6, p7, and p9 to HLA-DRB1*04:04. **c** Competitive binding of a biotin-labeled MIF peptide with an unlabeled MIF peptide or MIF variants with arginine or citrulline residues in p1, p4, p6, p7, and p9 to HLA-DRB1*04:05. **d** Competitive binding of a biotin-labeled myoglobin peptide with an unlabeled myoglobin peptide or myoglobin variants with arginine or citrulline residues in p1, p4, p6, p7, and p9 to HLA-DR3. Graphs depict the IC50 values (μM). *ND* non-detectable binding. Binding experiments were performed at least three times and plots show pooled experiments. The error bars show the standard error of the mean. ^*^Indicates a *p* value of <0.05

The genes encoding for HLA-DRB1, HLA-DQA1 and HLA-DQB1 are highly polymorphic and many different alleles have been identified in the human population. The residues within the HLA molecules involved in shaping peptide-binding pockets are known [6]. To select for HLA-DRB1 alleles that could potentially prefer citrulline over arginine residues, we made use of a MHC motif viewer [14], a web server that displays peptide-binding motifs for all HLA-DR alleles using a predictive algorithm. We searched for non-SE alleles that are common in Caucasians and that display a predicted preference for negatively charged amino acids. In this way, we selected HLA-DRB1*03:01 (abbreviated to HLA-DR3), one of the most common HLA-DR molecules in Caucasians, for further studies. HLA-DR3 was predicted to have a strong preference for negatively charged amino acids in pocket 4. As a control, the SE allele HLA-DRB1*04:04 and HLA-DRB1*04:05 were taken along to validate the experimental setup by comparing acquired data with published data. Figure 1a depicts the amino acid residues of HLA-DR3 and both HLA-DRB1*04 molecules involved in shaping the various binding pockets. Of all three HLA molecules, peptide-binding pocket 4 has a net positive charge, thereby explaining the predicted preference for negatively charged or neutral AA residues.

To systematically analyze if arginine-to-citrulline conversions of peptides enhanced the affinity for HLA-DRB1*04:04, HLA-DRB1*04:05, and HLA-DR3, we selected an ApoB, MIF, and myoglobin peptide respectively which were previously described to be accommodated by these alleles and for which the peptide-binding register is known [11, 15]. Next, we substituted each of the peptide positions interacting with peptide-binding pockets of the HLA molecule for arginine or citrulline residues. The effect of these substitutions was subsequently studied in peptide-binding assays. In this way, a systematic characterization of the ability of each of the peptide-binding pockets to accommodate arginine or citrulline residues was performed.

As depicted in Fig. 1b and c, HLA-DRB1*04:04 and HLA-DRB1*04:05 have a strong preference for citrulline over arginine in pocket 4, compatible with previously published data. Furthermore, it is apparent that also pockets 1, 6, and 9 of HLA-DRB1*04:05 prefer citrulline while pocket 7 does not distinguish between the two amino acids (Fig. 1c). On the other hand, as shown in Fig. 1d, HLA-DR3 was unable to accommodate either arginine or citrulline in pocket 4. Also for the other peptide-binding pockets, arginine-to-citrulline conversion did not result in enhanced peptide-binding affinities, whereas in pocket 6 and 9, arginine residues were preferred over citrulline.

Together, the data on the HLA-DRB1*04 molecules indicate that the peptide-binding assays provide a valid method to investigate amino acid preferences as pocket 4 prefers the accommodation of citrulline residues over arginine residues. However, this feature does not only apply to pocket 4, but also to other pockets accommodating anchor residues from the antigenic peptide. In contrast, the data indicate that HLA-DR3 does not have a preference for citrulline in pocket 4 or any of the pockets, while it prefers arginine residues as anchors in pockets 6 and 9.

### Presentation of arginine and citrulline residues by HLA-DQ molecules

**Fig. 2:**
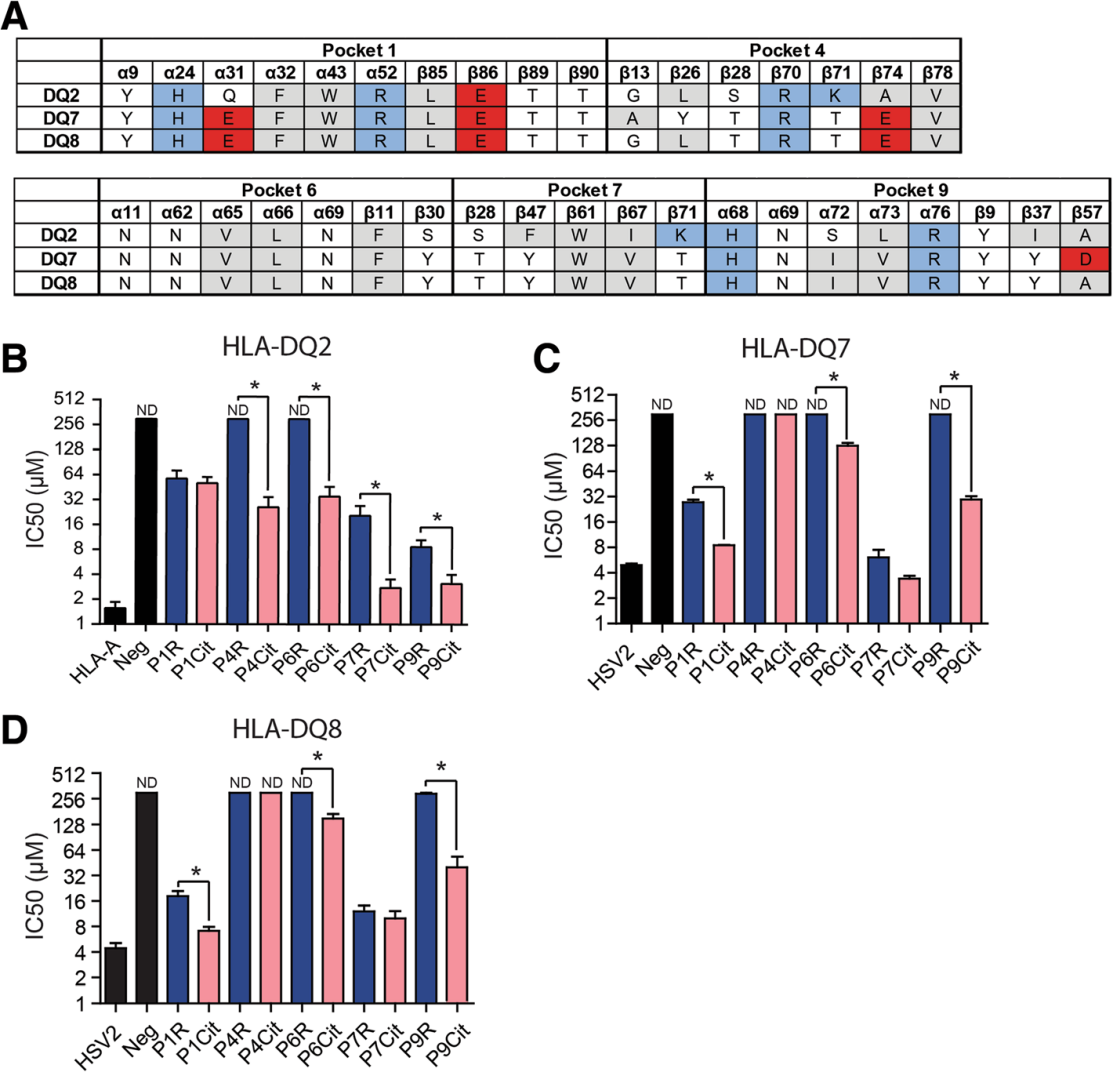
Accommodation of citrulline and arginine residues by HLA-DQ molecules. **a** Schematic representation of the differences in peptide-binding pockets between HLA-DQ2, HLA-DQ7 and HLA-DQ8. Amino acid (AA) residues are color coded according to their properties (*white* = hydrophilic, *gray* = hydrophobic, *red* = acidic, *blue* = basic). **b** Competitive binding of a biotin-labeled alpha-gliadin peptide with an unlabeled alpha-gliadin peptide or alpha-gliadin variants with citrulline or arginine residues in p1, p4, p6, p7, and p9 to HLA-DQ2. **c**–**d** Competitive binding of a biotin-labeled VP16 peptide with an unlabeled VP16 peptide or VP16 variants with citrulline or arginine residues in p1, p4, p6, p7, and p9 to HLA-DQ7 (**c**) and HLA-DQ8 (**d**). Graphs depict the IC50 values (μM). *ND* non-detectable binding. Binding experiments were performed at least three times and plots show pooled experiments. The error bars show the standard error of the mean. ^*^Indicates a *p* value of <0.05

Genes encoding for HLA-DR and HLA-DQ molecules are in tight linkage disequilibrium (LD) and inherit together in haplotypes. HLA-DQ molecules were recently implicated in the pathogenesis of RA [13]. The ability of HLA-DQ molecules encoded by predisposing and non-predisposing haplotypes to accommodate arginine and citrulline residues has not been extensively studied, although various peptides with varying anchor residues were tested on different HLA II molecules to determine the binding motif. Some HLA-DQ molecules were shown to prefer negatively charged amino acid residues, e.g., HLA-DQ2 (DQB1*02:01/DQA1*05:01), HLA-DQ7 (DQB1*03:01/DQA1*03:02), and HLA-DQ8 (DQB1*03:02/DQA1*03:01), each in distinct pockets or combinations of pockets. The latter two HLA molecules are particularly interesting as these are encoded by genes that are in tight LD with SE alleles and hence also associate with RA risk [13]. Figure 2a shows the amino acids shaping the various peptide-binding pockets of these HLA-DQ molecules.

To systematically analyze whether arginine-to-citrulline conversion of peptides enhanced their affinity for HLA-DQ molecules, we used a similar approach as applied for HLA-DR3. As the myoglobin peptide cannot be presented by these HLA-DQ molecules, we used two previously described ligands derived from MHC class I alpha antigen (for HLA-DQ2) and from herpes simplex virus VP16-protein (for HLA-DQ7 and HLA-DQ8) for which the binding register has been established [10, 12]. By using peptides that contained either an arginine or citrulline residue at defined anchor positions, we were able to show that all three HLA-DQ molecules preferred citrulline over arginine residues in different peptide-binding pockets of the HLA molecules. HLA-DQ7 (Fig. 2c) and HLA-DQ8 (Fig. 2d) preferred citrulline over arginine residues as anchors in peptide-binding pockets 1, 6, and 9, whereas HLA-DQ2 (Fig. 2b) molecules preferred citrulline residues in pockets 4, 6, 7, and 9, with a relatively high binding affinity for the latter two pockets.

**Fig. 3:**
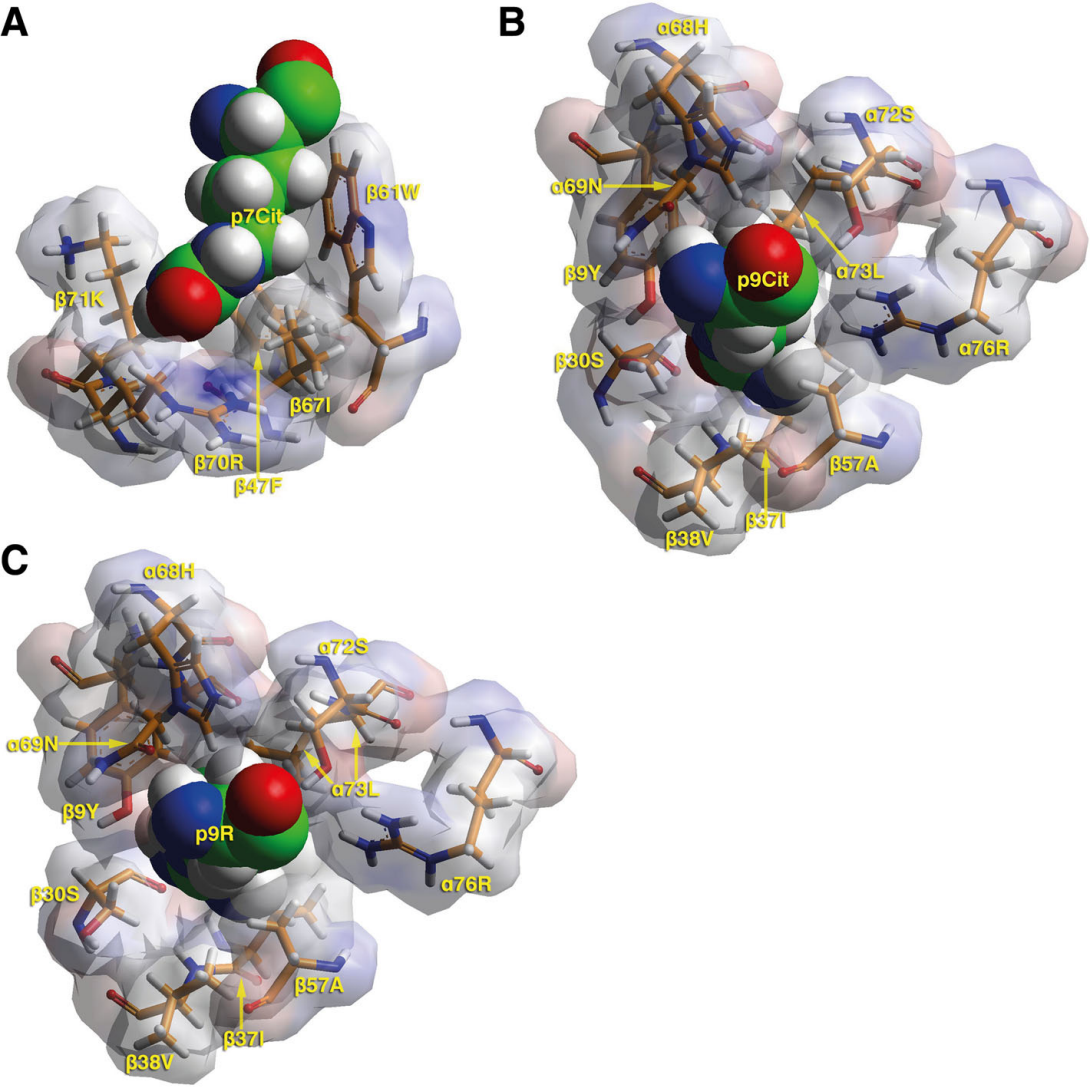
Model structures of citrulline residue accommodation in peptide-binding pockets 7 and 9 of HLA-DQ2. **a** T cell receptor (TCR)-view of the p7Cit anchor and the surrounding pocket 7 residues. The residues of pocket 7 are in van der Waals surface representation (partially transparent) with surfaces colored according to atomic charges (negative, *red*; neutral, *gray*; positive, *blue*). In addition, the HLA-DQ2 residues are shown in stick form (carbon, *orange*; oxygen, *red*; nitrogen, *blue*; hydrogen*, white*; sulfur, *yellow*). The p7 citrulline residue is depicted in space-filling form with the same color code as the stick form residues, except for carbon, which is in *green*. The figure is tilted with respect to the proper TCR view by +25 ° and +10 ° with respect to the x-axis and y-axis respectively. **b** TCR view of the p9Cit anchor and the surrounding pocket 9 residues. Colors and conventions as in A. Figure is tilted with respect to the proper TCR view by +5 ° and -10 °C with respect to the x-axis and y-axis respectively. **c** TCR view of the p9Arg anchor and the surrounding pocket 9 residues. Colors and conventions as in A. Figure is tilted as in B

It is evident from the peptide-binding experiments that the three HLA-DQ molecules tested are capable of binding citrulline-containing peptides. Molecular simulation was used in order to obtain a better appreciation of the molecular interactions facilitating the enhanced binding of citrulline-containing peptide used in the binding assays, in the context of HLA-DQ2. As is depicted in Fig. 3 and in line with the peptide-binding data, citrulline residues are predicted to be accommodated by both peptide-binding pocket 7 and 9 without any constraints in the surrounding residues or the anchor itself (Fig. 3a–b). Likewise, the model structure of arginine in pocket 9 predicts the accommodation of arginine within the pocket with only small rearrangements of the surrounding residues (Fig. 3c). The difference in binding affinity shown by the peptide-binding experiments is likely to be caused by the repulsion of the p9 arginine residue by the α76Arg in pocket 9.

Together these data demonstrate that an arginine-to-citrulline conversion can also enhance the peptide-binding affinity of peptides for several HLA-DQ molecules, thereby indicating that this property is not confined to HLA-DR-SE alleles. These data are important as they indicate that also HLA-DQ molecules could contribute to the induction of T cell immunity directed against citrullinated peptides, a mechanism thought to contribute to the autoimmune response underlying RA.

## Discussion

Over a decade ago, it was first demonstrated that arginine-to-citrulline conversion enhances the affinity of peptides for HLA-DRB1-SE alleles [7, 8]. It was suggested that this feature is unique to HLA-SE alleles and could thereby explain the HLA-RA connection. We have now analyzed HLA-SE and non-SE alleles and demonstrate that in addition to the HLA-DR-SE alleles, several HLA-DQ molecules also have the enhanced capacity to present citrullinated peptides as we show that peptides harboring a citrulline instead of an arginine in anchor positions display an enhanced affinity. Moreover, we show that not only pocket 4, but also other pockets within HLA-DRB*04 molecules show a preference for citrulline over arginine.

Determining the capacity to present citrullinated neo-epitopes for all different HLA molecules would require a large-scale approach given the fact that hundreds of different HLA-DR and HLA-DQ molecules have been described. However, the amino acid positions important for shaping peptide-binding pockets have been elucidated, thereby allowing the prediction of HLA-DQ and HLA-DR alleles that are likely to possess the ability to present citrullinated neo-epitopes. In this study, we focused on only a few SE-positive and SE-negative HLA class II molecules, present in relatively high frequency in the Caucasian population and that have not been studied before in this regard, and which are likely able to present citrullinated neo-epitopes. However, it is highly conceivable that also other HLA-DR or HLA-DQ molecules share similar capacities.

HLA-DRB1-SE alleles have been studied extensively with regard to their ability to present citrullinated peptides derived from antigens implicated in RA pathogenesis, for example peptides derived from vimentin and enolase [8]. For the majority of the SE alleles, it has been shown that these citrullinated peptides are accommodated in a more efficient fashion than the native forms [16, 17]. To the best of our knowledge, the capacity of HLA-DQ molecules to present citrullinated ligands has not yet been investigated. Interestingly, in a recent study, it was shown that T cells from HLA-DQ8 transgenic mice immunized with collagen type II (CII) respond better to citrullinated CII than to native CII, which could be explained by an enhanced capacity of HLA-DQ8 to present citrullinated neo-epitopes [18], though the binding was not shown in a direct approach. A limitation of our study is that we cannot formally rule out the possibility that our findings are influenced by the co-precipitation of HLA-DRB4 and HLA-DRB3 molecules as these molecules are co-expressed with HLA-DRB1*04 and HLA-DRB1*03 respectively. However, we anticipate that this influence, if present, is minimal because the binding registers of these HLA molecules differ substantially from the binding registers of HLA-DRB1*04 and HLA-DRB1*03. Moreover, the cell surface expression of HLA-DRB4 and HLA-DRB3 is considerably lower as compared to HLA-DRB1 molecules [19].

## Conclusions

The hypothesis that predisposing HLA molecules associate with ACPA-positive RA because of their capacity to present arginine-to-citrulline-converted epitopes with an enhanced affinity may not completely explain the molecular basis for the association between HLA-DR1-SE haplotypes and RA completely. It would, therefore, be interesting to better comprehend the additional contribution of the molecules encoded by the HLA-DRB1-SE haplotypes to seropositive RA. Together, this study provides a further refinement of the SE hypothesis and the possible contribution of citrulline-containing T cell epitopes in the pathogenesis of ACPA-positive RA via epitope presentation by non-SE HLA-DQ alleles.

## Abbreviations

AA: amino acid
ACPA: anti-citrullinated protein antibodies
ApoB: apolipoprotein B-100
CII: collagen type II
EBV: Epstein-Barr virus
FCS: fetal calf serum
HLA: human leukocyte antigen
HSV: herpes simplex virus
IMDM: Iscove’s modified Dulbecco’s medium
LD: linkage disequilibrium
MHC: major histocompatibility complex
MIF: macrophage migration inhibition factor
ND: non-detectable
RA: rheumatoid arthritis
SE: shared epitope
